# Accelerated first-in-human clinical trial of EIDD-2801/MK-4482 (molnupiravir), a ribonucleoside analog with potent antiviral activity against SARS-CoV-2

**DOI:** 10.1186/s13063-021-05538-5

**Published:** 2021-08-23

**Authors:** Wendy Holman, Wayne Holman, Stacy McIntosh, Wendy Painter, George Painter, Jim Bush, Oren Cohen

**Affiliations:** 1 Ridgeback Biotherapeutics, 3480 Main Highway, Unit 402, Miami, FL 33133 USA; 2 Emory University School of Medicine, Drug Innovations at Emory (DRIVE) and Emory Institute of Drug Development (EIDD), 954 Gatewood Road, Atlanta, GA 30329 USA; 3 Covance Clinical Research Unit Ltd., Springfield House, Hyde Street, Leeds, LS2 9LH UK; 4 Covance, Inc., 6 Drive, Research Triangle Park, Moore, NC 27709 USA

**Keywords:** COVID-19, SARS-CoV-2, Antiviral therapy, Ribonucleoside analog, Phase I, Accelerated start-up

## Abstract

A recently published article described the safety, tolerability, and pharmacokinetic profile of molnupiravir (Painter et al. 2021), a novel antiviral agent with potent activity against severe acute respiratory syndrome coronavirus 2 (SARS-CoV-2), the causative agent of coronavirus disease 2019 (COVID-19). Here, we report an unprecedented collaboration between sponsor, contract research organization (CRO), and regulatory authorities that enabled accelerated generation of these phase I data, including administration of the first-in-human (FIH) dose of molnupiravir within 5 days of receiving regulatory approval in the United Kingdom (UK). Single and multiple ascending dose (SAD and MAD, respectively) cohorts were dosed in randomized, double-blind, and placebo-controlled fashion, with a 6:2 active-to-placebo ratio in each cohort. A food-effect (FE) cohort included 10 subjects who were randomized to receive drug in the fasted or fed state followed by the fed or fasted state to complete a fed and fasted sequence for each subject. Dose escalation decisions were accelerated and MAD cohorts were initiated prior to completion of all SAD cohorts with the provision that the total daily dose in a MAD cohort would not exceed a dose proven to be safe and well-tolerated in a SAD cohort. Dosing in healthy volunteers was completed for eight single ascending dose (SAD) cohorts, seven multiple ascending dose (MAD) cohorts, and one food-effect (FE) cohort within approximately 16 weeks of initial protocol submission to the Research Ethics Committee (REC) and Medicines and Healthcare products Regulatory Agency (MHRA). Working to standard industry timelines, the FIH study would have taken approximately 46 weeks to complete and 33 weeks to enable phase 2 dosing. Data from this study supported submission of a phase 2/3 clinical trial protocol to the US Food and Drug Administration (FDA) within 8 weeks of initial protocol submission, with FDA comments permitting phase 2 study initiation within two additional weeks. In the setting of a global pandemic, this model of collaboration allows for accelerated generation of clinical data compared to standard processes, without compromising safety.

## Background

Coronavirus disease 2019 (COVID-19) was initially described as a new infectious respiratory illness in late 2019 [1–7]. A new pathogenic coronavirus, severe acute respiratory syndrome coronavirus 2 (SARS-CoV-2), was quickly shown to be the causative agent of COVID-19 [1, 5–7]. Clinical manifestations of COVID-19 range from asymptomatic infection to pneumonia to acute respiratory distress syndrome (ARDS), cytokine storm, coagulopathy, thrombosis, and a number of other extrapulmonary syndromes [4, 8–10]. In some instances, an otherwise benign disease course can very rapidly deteriorate during the second week of illness, often requiring supplemental oxygen therapy and intensive care [4, 9]. Although aberrant immune responses may drive some COVID-19 pathology, it is clear that a high level of viral replication early during the course of disease is associated with disease severity [11, 12]. Thus, there is an urgent need for a potent antiviral agent that does not require parenteral administration and, therefore, can be given early in the course of disease. Given the current understanding of COVID-19 pathogenesis, such a strategy involving early potent antiviral therapy should have a high probability of success.

Molnupiravir is the orally bioavailable 5′-isobutyrate prodrug of a direct-acting antiviral ribonucleoside analog, β-D-N4-hydroxycytidine, or EIDD-1931. Molnupiravir is cleaved in the plasma to liberate EIDD-1931. EIDD-1931 is phosphorylated intracellularly to its corresponding 5′-triphosphate, the active antiviral agent, by host kinases [13, 14]. EIDD-1931 inhibits replication of multiple RNA viruses, and its antiviral activity was verified in animal models of influenza, various coronaviruses, respiratory syncytial virus (RSV), Venezuelan equine encephalitis virus (VEEV), Chikungunya, and Ebola virus infection [15–18].

Similar to other nucleoside analog antiviral agents, EIDD-1931 5′-triphosphate acts as a competitive alternative substrate for the virally encoded RNA-directed RNA polymerases (RdRp) and is incorporated into nascent chain RNA. Since the N4-hydroxycytosine base of EIDD-1931 can tautomerize, EIDD-1931 can substitute for either cytidine or uridine and pair with either guanosine or adenosine, respectively, thereby disrupting the fidelity of viral genomic replication [19]. This results in an accumulation of mutations that increases with each cycle of viral replication. The process whereby the mutation rate is increased by exposure to a drug is referred to as viral decay acceleration and results in viral ablation [20]. This mechanism of action for EIDD-1931 has been validated through demonstration of activity against Middle East respiratory syndrome coronavirus (MERS-CoV), VEEV, and influenza A virus (IAV) [15, 18, 21, 22].

When SARS-CoV-2 emerged as a global health threat in late 2019, an Investigational New Drug (IND) application for molnupiravir was being prepared for the treatment of IAV. At that time, there were in vitro data demonstrating that EIDD-1931 was active against other pathogenic coronaviruses [15]. It was quickly demonstrated that EIDD-1931 had potent antiviral activity against SARS-CoV-2 in a human lung epithelial cell line and against a clinical isolate of SARS-CoV-2 (2019-nCoV/USA-WA1/2020) in African green monkey kidney (Vero) cells [22]. In primary human airway epithelial cell cultures, which recapitulate the architecture and cellular complexity of the conducting airway, EIDD-1931 also demonstrated potent, dose-dependent in vitro antiviral activity against severe acute respiratory syndrome coronavirus (SARS-CoV), MERS-CoV, and SARS-CoV-2 [22].

Although nucleoside analogs are important components of modern antiviral therapy, their activity can be markedly reduced by a single or small number of viral mutations. The structural motifs of the RdRp are highly conserved among coronaviruses, including SARS-CoV-2. Resistance to another broad spectrum ribonucleoside analog, remdesivir, is mediated by mutations in RdRp residues 480 (F480L) and 557 (V557L) in SARS-CoV and in a murine coronavirus, mouse hepatitis virus (MHV) [23]. Of note, these two remdesivir resistance mutations, alone or in combination, actually conferred increased sensitivity to inhibition by EIDD-1931 [22].

In a mouse model of SARS-CoV infection, administration of molnupiravir beginning 2 h before or 12 or 24 h after infection demonstrated robust antiviral activity and amelioration of disease (including parameters such as body weight, viral load in lung tissue, and lung hemorrhage), over a 5-day observation period [22].

The phase I clinical trial data describing the safety, tolerability, and pharmacokinetic profile of molnupiravir were recently published [24]. This paper describes how these data were generated along a significantly accelerated timeline.

## Rapid initiation of a clinical research program to study molnupiravir as a treatment for COVID-19

### Start-up activities

Following an initial call with the sponsor, the contract research organization (CRO) immediately assembled a project team composed of clinical pharmacology, operations, medical, pharmacy, bioanalytical, data management, biostatistics, medical writing, project management, pharmacokinetics, and regulatory staff (project day 1). A joint sponsor-CRO project team developed an expedited phase 1 single ascending dose (SAD) and food-effect (FE) trial design, which included a placeholder for the later addition of multiple ascending dose (MAD) cohorts. This approach facilitated rapid startup and reduced the time to acquire MAD cohort data at physiologically relevant doses (based on animal model data [17]) to 40 calendar days from project initiation.

In order to maximize the likelihood of completing the phase 1 study without interruption due to possible COVID-19-related closure of investigative sites, regulatory submissions in the USA and UK were pursued in parallel. In the USA, a recently submitted molnupiravir IND for the treatment of IAV provided the foundation for submission of preliminary data on the drug’s activity against pathogenic coronaviruses. The Food and Drug Administration (FDA) expedited the conduct of a Pre-IND Meeting and review of the SARS-CoV-2 IND (cross-referencing the IAV IND). The IAV IND content plus the SARS-CoV-2 data were the basis for submission packages in the UK. An Expert Working Group for COVID-19 was established by the UK Commission on Human Medicines, and the Medicines and Healthcare products Regulatory Agency (MHRA) published a guidance on Clinical Trial Applications (CTAs) for COVID-19 products, which specified the procedures to supply rapid scientific advice, review, and approval for potential COVID-19 treatments [25]. MHRA confirmed that the CTA should include all the usual components (i.e., Investigator’s Brochure, Study Protocol and Investigational Medicinal Product Dossier), but that review of final draft documents would occur on a rolling basis. Review comments were provided by the MHRA in real-time, permitting the project team to make requested changes prior to formal CTA submission. The MHRA further advised that Research Ethics Committee (REC) submission should not proceed in the usual fashion, via the Combined Ways of Working pathway (which allows consecutive regulatory and ethics review from a single application), but rather a request for expedited review should be made through the Health Research Authority (HRA) Director of Approvals Service. With regard to the protocol, the MHRA advised that the SAD design should be fully defined in the initial submission, but that subsequent components (e.g., MAD cohorts) could be referenced as placeholders for tailoring at a later date. Protocol amendments received similar expedited review by both MHRA and the REC.

Over the first 11 days of the project, all MHRA regulatory submission documents were completed and the SAD study protocol was finalized, culminating in submission to the REC on day 12 of the project. The REC convened the following day, with the principal investigator (PI) in attendance by telephone. Provisional REC approval was granted on day 16 of the project. On that same day, formal CTA submission was achieved. Full REC approval was received on day 18 and CTA approval on day 19 of the project, respectively. This enabled the screening of healthy volunteers to start on day 20 and the first human dose to be administered on day 23 of the project, respectively.

In parallel, the CRO pharmacy prepared for supply of the investigational medicinal product in accordance with Good Manufacturing Practices. In order to further expedite study start-up, a decision was made to dose SAD cohorts with a powder-in-bottle preparation which was used for extemporaneous preparation of a solution for oral administration; a capsule preparation was later introduced for MAD cohorts through CTA amendment. Active Pharmaceutical Ingredient (API) was received by the CRO pharmacy on day 17 of the project. On day 18, API samples were sent to a contract laboratory for confirmatory identification (ID) testing. ID testing and the technical batch run were both completed on day 19. Pharmacy setup activities, which routinely require two to three months to perform, were completed within seven calendar days. Bioanalytical setup was also completed very rapidly, with plasma method development work and core plasma validation experiments completed in 2 weeks, rather than the usual 5 weeks.

### Protocol design

The initial protocol design (CTIN NCT04392219) included two parts: a randomized, double-blind, placebo-controlled SAD study of three cohorts (part 1) and a FE cohort (part 2). The protocol was written to include up to four additional cohorts in part 1 and one additional cohort in part 2. SAD study cohorts included eight subjects each randomized 6:2 to molnupiravir and placebo, respectively. The initial SAD cohort included dosing of two sentinel subjects, each one assigned to either molnupiravir or placebo. The safety results for the sentinel group were reviewed 24 h post-dose, prior to dosing the remainder of the subjects in cohort 1. Following dosing on study day 1, subjects remained in the clinic through completion of study assessments on study day 4. Subjects returned to the clinic on study day 9 for assessments and study day 15 for the end of study (EOS) visit.

For part 2, the effect of food on molnupiravir pharmacokinetic (PK) parameters was examined in a single cohort of 10 healthy volunteers. Five subjects were randomized to receive drug in the fed then fasted state (sequence 1), and five subjects were randomized to receive drug in the fasted then fed state (sequence 2), with an intervening washout period in the middle of each sequence. Treatment was administered in open-label fashion. In each sequence, subjects were discharged from the clinic on the fourth day after dosing and returned to the clinic on the ninth day after dosing for blood draw and assessments. Subjects returned on the fourteenth day after dosing for check-in to begin the second dosing sequence (fasting if sequence 1 was in the fed state, and fed if sequence 1 was in the fasting state) on study day 15.

Upon initiation of part 1 (day 23), a protocol amendment to add a MAD component (part 3) was submitted to the MHRA and REC on days 23 and 24, respectively. Again, a final draft protocol was informally submitted to MHRA and comments were provided in real-time prior to formal submission of the substantial amendment. This enabled MHRA and REC approvals 2 days after formal submission, on days 25 and 26, respectively. A comparable IND amendment was submitted in parallel to FDA for US sites as a back-up. Part 3 was a randomized, double-blind, placebo-controlled MAD study with three initial cohorts and the option to include up to four additional cohorts. Cohorts in the MAD study included eight subjects each, randomized 6:2 to molnupiravir and placebo, respectively. Subjects were dosed twice daily on study days 1–5 and received a further single dose on the morning of study day 6. Subjects remained in the clinic through completion of study assessments on study day 9. Subjects returned to the clinic on study day 15 for assessments and on study day 20 for the end of study visit. The protocol allowed part 3 to run in staggered parallel fashion with part 1, provided that the total daily dose administered in part 3 did not exceed a dose already shown to be safe and well-tolerated in part 1.

In parts 1 (SAD) and 3 (MAD), dose escalation proceeded based upon satisfactory review by the PI, Medical Monitor, and sponsor of the blinded safety and tolerability data collected up to 72 h post-dose for a preceding dose cohort. The 72-h period was established based on the anticipated drug clearance. Plasma PK data from the lower dose levels were also reviewed as they became available. Dose escalation could only occur if data from a minimum of six subjects in total and a minimum of four subjects receiving molnupiravir were used to make the dose escalation decision. The dose levels tested in later MAD cohorts were selected so as not to increase the dose given by more than 3-fold for predicted non-pharmacologically active dose levels and by 2-fold for predicted pharmacologically active dose levels.

### Execution of the study

The CRO volunteer recruitment team initiated contact with healthy volunteers registered in the CRO recruitment database with interest in participating in clinical trials before the CTA was approved. Volunteer screening commenced on day 20 of the project, 1 day after CTA approval was granted, and approximately 80 screening appointments were booked to occur between days 20–22 of the project, over a public holiday weekend in the UK. The first-in-human (FIH) dose was administered on day 23 of the project, just 15 business days after initial contact between sponsor and CRO. Rapid compilation of full safety information, including cohort characteristics, vital signs, electrocardiograms, complete blood counts with differential, clinical chemistry panels, urinalyses, and adverse event listings, was required while adhering to MHRA data quality standards. Dose escalation meetings were held on the day of cohort discharge irrespective of the day of the week. Subjects for a subsequent cohort were screened and brought to the clinic such that their dosing could proceed on the day following safety review, provided all dose escalation criteria were met.

The first three SAD cohorts were dosed on day 23/24 (sentinel group/main group), day 28, and day 32 of the project. On day 32 of the project, the first MAD group was also dosed. Review of data from the first three cohorts of part 1 (SAD) indicated favorable PK and an absence of any safety or tolerability issues. Therefore, the decision was made to enroll cohorts at higher dose levels in part 1 than initially planned, in order to determine if any dose-limiting safety or tolerability issues would emerge. In addition, PK modeling suggested that higher clinical dose levels may be necessary; it was therefore necessary to study higher dose SAD cohorts to fully explore the therapeutic dose range. Following the SAD Cohort 3 dose escalation meeting, intensive communication with MHRA Medical Assessor enabled a meeting within days that included review of a summary rationale document, as well as the safety and PK data. The Medical Assessor then conferred with the Nonclinical Assessor and agreed with the proposed rationale. A protocol amendment was submitted to MHRA and the REC, and both approvals were received the following day (day 40 of the project), just 1 week from the time of the SAD Cohort 3 dose escalation meeting. Consistent with contingency plans noted above, submission to FDA occurred during the same week.

The second MAD cohort was dosed on day 41 of the project. SAD cohorts 4 and 5 were dosed on days 45 and 49 of the project, respectively. Review of these data provided the necessary safety clearance to dose the MAD cohorts 3 and 4 on days 50 and 59, respectively. The food effect study (part 2) was completed from days 35–68 of the project.

The dose level administered in MAD cohort 3 was selected as the initial dose level to be administered to patients in phase 2. Molnupiravir was therefore positioned for phase 2 studies within approximately 8 weeks of protocol submission. On day 50 of the project, the sponsor submitted a phase 2/3 protocol to FDA for a study of molnupiravir in patients with symptomatic COVID-19 not requiring hospitalization. FDA comments permitting study initiation were received on day 66 of the project, in turn enabling initiation of a pivotal trial following dose finding in the phase 2 studies.

SAD cohort 6, which received the increased maximum dose level allowed by the protocol, was dosed on day 60 of the project. Another meeting with the MHRA assessor transpired on day 73 of the project to provide rationale for a further increase to the maximum dose level allowed by the protocol, and a second substantial amendment was submitted to MHRA (day 87) and REC (day 88) with approvals received by day 95. MAD cohort 5 was successfully dosed on day 84 of the project, while waiting for MHRA and REC approval. Following approval, SAD cohorts 7 and 8 were dosed on days 100 and 114, respectively, and MAD cohorts 6 and 7 on days 105 and 121, respectively.

**Fig. 1:**
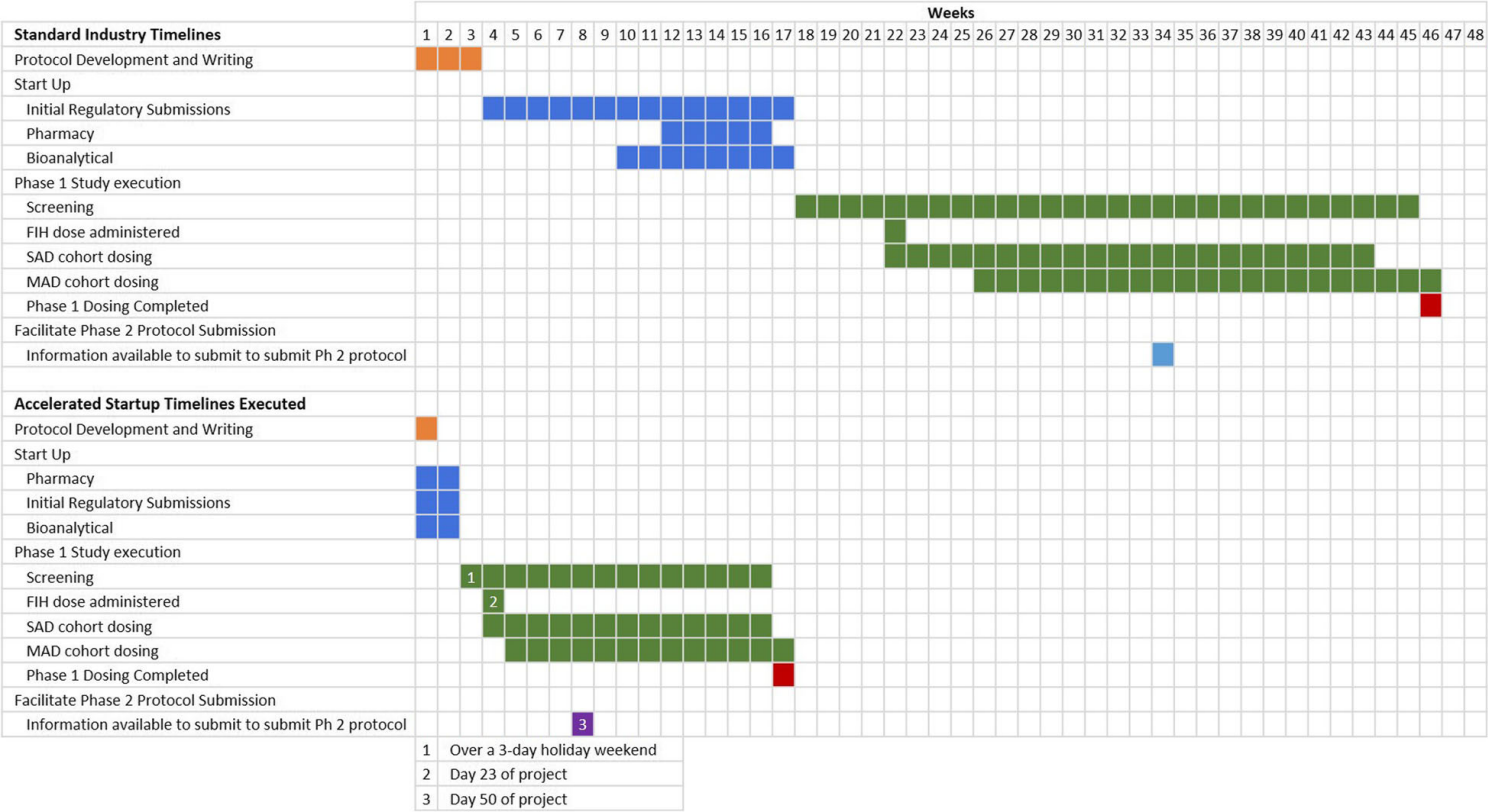
Time from submission of the protocol to the ethics committee and regulatory authority to completion of dosing of 8 SAD, 7 MAD, and 1 FE cohorts under standard industry timelines compared with what was achieved under accelerated conditions. SAD, single ascending dose; MAD, multiple ascending dose; FE, food effect; FIH, first in human

Dosing of the final cohort of subjects in the phase 1 program was completed approximately 16 weeks from the time of initial protocol submission for review by the regulatory authority and REC. Working to standard industry timelines, the FIH study would have normally taken approximately 46 weeks to complete and 33 weeks to enable phase 2 dosing (Fig. 1).

## Conclusion

The speed with which this clinical program was accelerated is due to the extraordinary collaboration between sponsor, CRO, and regulatory agencies, as well as the efficient study conduct made possible by the work of many individuals and by coordinated collaboration between stakeholders. Key success factors included alignment of all stakeholders with regard to a sense of urgency, execution of study elements in parallel wherever possible, acceleration of data processing and review, and contingency planning. Within eight weeks of phase 1 protocol submission, data were available that enabled dose selection for a phase 2 protocol for this promising new antiviral ribonucleoside analog. Had this study been conducted to standard industry timelines, it would have taken 8–9 months to generate the data necessary to enable a phase 2 study. Indeed, phase 2 studies have now commenced (ClinicalTrials.gov identifier NCT04405570 and NCT04405739), and study NCT04405570 completed in February, 2021. This case study demonstrates that urgent, coordinated efforts to support expedited study start-up and execution, including collaboration between sponsor, CRO, and regulatory authorities, can greatly accelerate early clinical development of promising drug therapies under extraordinary circumstances, such as the SARS-CoV-2 pandemic [26].

## Data Availability

Refer to clinical trial master file (reference [23]; ClinicalTrials.gov identifier NCT04392219.

## Abbreviations

API: Active pharmaceutical ingredient
ARDS: Acute respiratory distress syndrome
COVID-19: Coronavirus infectious disease 2019
CRO: Contract research organization
CTA: Clinical trial application
EOS: End of study
FDA: Food and Drug Administration
FE: Food effect
FIH: First-in-human
HRA: Health Research Authority
IAV: Influenza A virus
IND: Investigational new drug
MAD: Multiple ascending dose
MERS-CoV: Middle East respiratory syndrome coronavirus
MHRA: Medicines and Health products Regulatory Authority
MHV: Mouse hepatitis virus
PI: Principal investigator
RdRp: RNA-dependent RNA polymerase
REC: Research ethics committee
RSV: Respiratory syncytial virus
SAD: Single ascending dose
SARS-CoV: Severe acute respiratory syndrome coronavirus
SARS-CoV-2: Severe acute respiratory syndrome coronavirus 2
UK: United Kingdom
VEEV: Venezuelan equine encephalitis virus
